# Effect of a traditional Japanese garlic preparation on blood pressure in prehypertensive and mildly hypertensive adults

**DOI:** 10.3892/etm.2012.819

**Published:** 2012-11-20

**Authors:** YASUSHI NAKASONE, YOSUKE NAKAMURA, TETSURO YAMAMOTO, HIDEYO YAMAGUCHI

**Affiliations:** 1 Kenkoukazoku, Inc., Kagoshima 892-0848;; 2 Medical Corporation Keiaikai Nakamura Hospital, Oita 874-0937;; 3 Research Center, Total Technological Consultant Co., Ltd., Tokyo 150-0021, Japan

**Keywords:** garlic, traditional Japanese garlic homogenate-based supplementary diet, blood pressure, mild hypertension, prehypertension

## Abstract

Numerous clinical studies have used differing garlic preparations leading to controversial results with regard to the hypotensive effect of garlic. This randomized, double-blind, placebo-controlled study was designed to determine the effect of a traditional Japanese garlic homogenate-based supplementary diet (GH diet) product on blood pressure (BP) in subjects with prehypertension and in those with mild hypertension. In total, 34 eligible subjects with prehypertension and 47 with mild hypertension were treated with a daily dose of GH diet (300 mg as dried garlic homogenate; n=16 and 23, respectively) or placebo (n=18 and 24, respectively) for 12 weeks. Of these, 32 prehypertensive subjects (15 on the GH diet and 17 on the placebo) and 40 mildly hypertensive subjects (19 on the GH diet and 21 on the placebo) completed the study and were subjected to efficacy analyses. Systolic and diastolic BPs were monitored at weeks 4, 8 and 12 during the treatment and at post-week 4 following the termination of the treatment. The GH diet induced significant reductions of systolic BP (of between 6.6 and 7.5 mmHg) and diastolic BP (of between 4.6 and 5.2 mmHg) compared with the placebo subsequent to 8 and 12 weeks of treatment. A 12-week intake of the GH diet did not cause any clinically problematic side-effects. We conclude that the GH diet was well tolerated, and had a clinically relevant hypotensive effect in adults with mild hypertension, but not in those with prehypertension.

## Introduction

Hypertension, defined as a systolic blood pressure (BP) >140 mmHg and/or a diastolic BP >90 mmHg, is one of the major risk factors for various cardiovascular morbidities, including stroke, coronary heart disease and kidney dysfunction, as well as for mortality (1). Although hypertension affects up to 30% of the adult population in the majority of countries (2), >50% of hypertensive individuals are unaware of their condition (3). Prehypertension, formerly termed borderline hypertension or high-normal BP, is usually defined as a systolic BP of 130–139 mmHg and a diastolic BP of ≤89 mmHg, or as a systolic BP of ≤139 mmHg and a diastolic BP of 85–89 mmHg (2,4). This condition is known to be a precursor of hypertension (4,5) and is associated with excess morbidity and mortality from cardiovascular causes (4,6). Thus, lowering BP may be beneficial not only for the hypertensive population but also for the prehypertensive population. For the initial management of high BP, relevant lifestyle modifications, including ensuring optimal nutrition, weight reduction and regular physical activity, were established by The Japanese Society of Hypertension (JSH) in 2004 (7). In recent years, food supplementation, mainly using natural foods or their components, has been considered to provide another, less strenuous non-pharmacological option for lowering BP, particularly for individuals with borderline to mildly high BP that does not warrant the prescription of antihypertensive drugs.

Garlic (*Allium sativum*) has a long history of use as a foodstuff and as a pungent spice in numerous countries. Garlic has also been used in a number of cultures for various medicinal purposes. Previous pharmacological studies concerning garlic have revealed that it has various prophylactic and remedial properties beneficial to good health, among which its hypotensive activities are the most notable (8–11). Over the last 30 years, numerous clinical studies have been carried out to examine the effects on health of using various garlic-based products containing dried garlic powder, garlic oil or garlic extract as the basal component. Such varied types of garlic preparation may contain widely varying types of sulfur-containing phytochemicals due to the different methods of producing the preparations. This may precipitate varying biological responses in humans. Although several clinical trials have suggested that garlic lowers systolic and/or diastolic BP, negative results have been obtained in a larger number of trials, as recognized by several systematic reviews and meta-analyses (8,12–15). These studies may have been complicated by differences in study design, study population, dosage, duration of treatment or type of garlic preparation.

In Japan, a unique type of garlic preparation has been used for centuries as a traditional health food. This is a garlic homogenate-based supplementary diet (GH diet) that is made by kneading and pulverizing crushed garlic together with egg yolk. A considerable number of commercial products containing the GH diet are currently available on the market. Tradition dictates that the egg yolk is included to enrich the nutrient content and alleviate the untoward effects of garlic, including gastrointestinal complaints and breath and body odor. Using a representative GH diet product, we have previously demonstrated that chronic oral administration significantly lowered BP in spontaneously hypertensive rats (16). The promising results from this animal study tempted us to conduct a clinical trial to evaluate the effects of short-term supplementation with the same GH diet product on the systolic and diastolic BPs of adult subjects with prehypertension or mild hypertension.

## Subjects and methods

### Subjects and eligibility

Two populations of adult male and female Japanese participants aged 20–70 years old were included in this study: one population comprised prehypertensive individuals with systolic BPs between 130 and 139 mmHg or diastolic BPs between 85 and 89 mmHg, and the other contained mildly hypertensive (stage I hypertensive) individuals with systolic BPs between 140 and 159 mmHg or diastolic BPs between 90 and 99 mmHg (as defined according to the criteria of the 2004 JSH Guidelines for the Management of Hypertension) (7). Subjects were excluded if they were receiving antihypertension treatment or other medications that may have affected their BP, or if they currently suffered from diabetes, chronic renal failure or cardiovascular dysfunction. They were also excluded if they had a past history of such medical conditions or routinely consumed alcohol in a daily dose of ≥60 g, or if they had secondary hypertension, white-coat hypertension or known allergies to garlic or any other ingredients of the GH diet or placebo. The other exclusion criteria were participation in another clinical study at the start time of the present study, being a pregnant woman, nursing mother or a woman of childbearing potential or the presence of any clinically significant medical condition judged by the investigator to preclude the participant's inclusion in the study. Written informed consent was obtained from all participants prior to their enrollment in the study.

The study participants attended 2 screening visits (clinic visits 1 and 2) at an interval of 2 weeks, each of which included medical and life-style histories, physical examinations, laboratory tests and measurements of BP. The study treatment began ∼2 weeks later (clinic visit 3; baseline). Participants included in the prehypertensive and mildly hypertensive populations were those whose systolic and diastolic BPs, measured at visits 1 and 2, maintained levels within the ranges defined for prehypertension and mild hypertension, respectively. All participants were further instructed to self-measure their BP at home during the 2-week interval between clinic visits 2 and 3, and were again confirmed to have a systolic BP of 130–139 mmHg and/or a diastolic BP of 85–89 mmHg for prehypertensive subjects and a systolic BP of 140–159 mmHg and/or a diastolic BP of 90–99 mmHg for mildly hypertensive subjects.

### Study design and study diet

A randomized, double-blind, placebo-controlled study was designed to assess the efficacy and safety of the GH diet for lowering the BP in enrolled subjects when compared with the placebo. The study consisted of a run-in period of 4 weeks for screening and randomization of eligible subjects, a 12-week treatment period and a 4-week post-treatment follow-up period. The study mainly occurred between September 2006 and May 2007 at the Medical Corporation Keiaikai Nakamura Hospital (Beppu, Japan). The study protocol was approved by the Institutional Review Board of the hospital. The study was conducted in accordance with the principles of the Declaration of Helsinki in 1995 (as revised in Edinburgh, 2000) and the Ethical Guidelines for Epidemiological Research (2004) enacted by the Japanese Government in 2004.

The GH diet used in this study was contained in a 500 mg capsule ‘Dentou-ninniku-ranwo™’ (Kenkoukazoku Inc., Kagoshima, Japan). This capsule contained 188 mg of a garlic preparation consisting of a powdery mixture of garlic homogenate and egg yolk (as the active ingredient). This is made by kneading and pulverizing crushed garlic bulbs together with egg yolks at a weight ratio of 80:20. The capsule also contained 266.5 mg rapeseed oil (as the solvent) and 45.5 mg beeswax (as the stabilizer). Placebo capsules contained dextrin, rapeseed oil and beeswax. The contents were colored by saffron and caramel to make them similar in appearance to the contents of the active capsule.

Throughout the course of the study, from the run-in period to the post-treatment follow-up period, each participant was required to keep a study diary of their allocated capsule intake, any adverse events experienced, dietary composition, physical activity assessed by a passometer and all medications or therapies received. Participants were also instructed to maintain their body weight and to avoid exercising, eating or drinking in excess of the levels of their usual habits. Upon completion of the run-in period, eligible subjects with prehypertension and those with mild hypertension were sequentially assigned to either one of the two masked study capsules (GH diet or placebo) according to a predetermined computer-generated randomization schedule. The participants were instructed to take 2 capsules (300 mg as dehydrated GH) per day at any time of the day. Taking <85% of the prescribed course of the allocated study capsule was considered as non-compliance with the treatment. Such non-compliant subjects were excluded from the efficacy assessment.

To assess efficacy and safety, medical inspections, measurements of BP and other physical parameters and laboratory tests were performed at baseline (clinic visit 3), at 4 weeks (clinic visit 4), 8 weeks (clinic visit 5) and 12 weeks (clinic visit 6) following the start of the treatment, and also after 4 weeks subsequent to the termination of the treatment (clinic visit 7; post-week 4).

### BP measurement

Systolic and diastolic BPs were measured from the left arm using an automated sphyngomanometer (HEM-7051T; Omron Corp., Kyoto, Japan). Measurements were performed repeatedly (a maximum of 5 times) at 2-min intervals following a 10-min or longer rest in a sitting position, until the variance of 2 successive measurements was ≤5 mmHg. The mean values of 2 such measurements were then used as an estimate of BP.

### Blood sampling and laboratory tests

Fasting blood samples were collected by venopuncture at each clinic visit. The EDTA tubes were then refrigerated immediately and centrifuged within 2 h. Plasma samples were stored in a frozen state until analysis. All plasma samples were analyzed for routine hematological and biochemical parameters.

### Assessments of diet and physical activity behaviors

Food intake and exercise behaviors were assessed at every clinic visit based on the daily records from the study diary. The exercise assessment was aided by the use of a passometer. Data were analyzed at a group level.

### Masking

Subjects and all study personnel (including the data analyst) were blinded to the treatment assignments throughout the study. Placebo capsules were similar in appearance and size to the active capsules. Blinding of the subjects, effectiveness of the blinding and the tolerance of the study capsules were assessed at each clinic visit by analyzing the study diary. Upon completion of the study, subjects were asked if they knew which of the two treatments they were assigned to.

### Safety assessments

Safety parameters were the incidence and severity of treatment-related adverse events that the subjects reported that they had experienced throughout the treatment and post-treatment follow-up periods.

### Statistical methods

Efficacy and safety were assessed on the basis of data from the per-protocol-based (PPB) and the intention-to-treat (ITT) populations, respectively. The PPB population consisted of the subjects with prehypertension and those with mild hypertension who completed all clinic visits at the prescribed times and the full protocol and who were suitably compliant with the prescribed regimen (GH diet or placebo), with an overall compliance rate >85%. The ITT population included all subjects in the study populations who were randomized and received at least 1 dose of the assigned study capsules and for whom any follow-up evaluation (physical parameters, laboratory tests, adverse events and self-reported comments) were obtained. Thus, the ITT population was to include those subjects who dropped out of the study, were removed or lost prior to follow-up or were non-compliant with the regimen specified in the protocol.

The baseline characteristics of the randomized subjects with prehypertension and those with mild hypertension were compared between the GH and placebo groups using the Student's unpaired t-test for data expressed as mean ± standard deviation (SD), and by the Chi-square test for category variables. The efficacy results were expressed in terms of mean ± standard error (SEM). The Bonferroni method was used for within-group efficacy assessments, and the Student's unpaired t-test was used for comparison of the treatment effects between the two groups. P<0.05 was considered to indicate a statistically significant difference.

## Results and discussion

### Characteristics of subjects

In total, 81 subjects, consisting of 34 with prehypertension and 47 with mild hypertension, were enrolled in the study and assigned to either the placebo (n=18 and 23, respectively) or GH diet groups (n=16 and 24, respectively). A total of 80 subjects completed all clinic visits resulting in a retention rate of 99%. One male subject (mild hypertension, placebo) dropped out for an unrelated personal reason after 4 weeks of treatment. In addition, 8 subjects were also excluded from efficacy analysis for the following reasons: 6 subjects delayed taking the assigned study capsules for ≥4 days following visit 3 (1 mild hypertension, placebo; 1 prehypertension, GH; and 4 mild hypertension, GH); 1 subject missed taking the assigned study capsules for >4 days (prehypertension, placebo) and 1 subject was receiving analgesic medication (mild hypertension, placebo). As a result, 32 prehypertensive subjects (17 in the placebo group and 15 in the GH group) and 40 mildly hypertensive subjects (21 in the placebo group and 19 in the GH group) were subjected to efficacy analysis.

Table I shows the baseline characteristics of the randomized subjects with prehypertension or mild hypertension who were assigned to either the placebo or the GH diet. In each subject population there were no significant differences between the placebo and GH groups. Table I also demonstrates that the mean baseline levels of all physical and biochemical parameters, with the exception of systolic and diastolic BPs, for the two groups of mildly hypertensive subjects were within the normal ranges for clinical measurements.

### Effect on systolic and diastolic BPs

**Figure 1.**
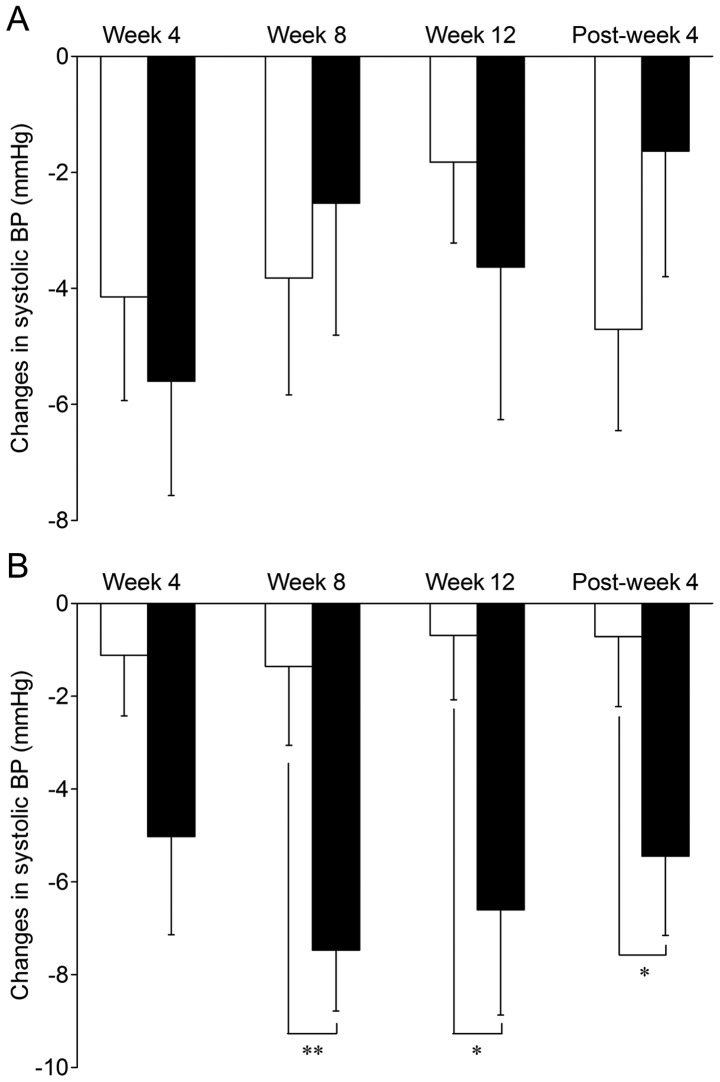
Changes from the baseline systolic BP following treatment at weeks 4, 8 and 12 and the following treatment at post-week 4 in (A) the placebo group (n=17) and GH group (n=15) of prehypertensive subjects and in (B) the placebo group (n=21) and GH group (n=19) of mildly hypertensive subjects. ^*^P<0.05, ^**^P<0.01 against placebo value. White bars represent the placebo groups, and black bars represent GH groups. GH, garlic homogenate-based supplementary diet; BP, blood pressure.

The changes in the mean value of the systolic BP over the 12-week treatment period and the subsequent 4-week post-treatment follow-up period are shown in Table II. In prehypertensive subjects, systolic BP was not consistently modified by the GH diet treatment. Although there was a significant reduction from the baseline figure at week 4 of GH diet treatment (3.4%, P<0.05), reductions of a similar extent (3–4%, P<0.05 each) were also observed in the placebo group at week 4 and post-week 4. By contrast, in mildly hypertensive subjects, the GH diet treatment significantly reduced systolic BP at week 4 (3.5%; P<0.05), week 8 (5.3%; P<0.01) and week 12 (4.6%; P<0.01), as well as at post-week 4 (3.9%; P<0.05), while no such significant reduction was observed at any time following the intake of the placebo. As shown in Fig. 1, there were significant differences between the GH and placebo groups of mildly hypertensive subjects at week 8 (mean change from baseline: GH, −7.5±1.3 mmHg; placebo, −1.4±1.7 mmHg; P<0.01) and week 12 (mean change from baseline: GH, −6.6±2.3 mmHg; placebo, −0.7±1.4 mmHg; P<0.05), as well as at post-week 4 (mean change from baseline: GH, −5.4±1.7 mmHg; placebo, −0.7±1.5 mmHg; P<0.05).

**Figure 2.**
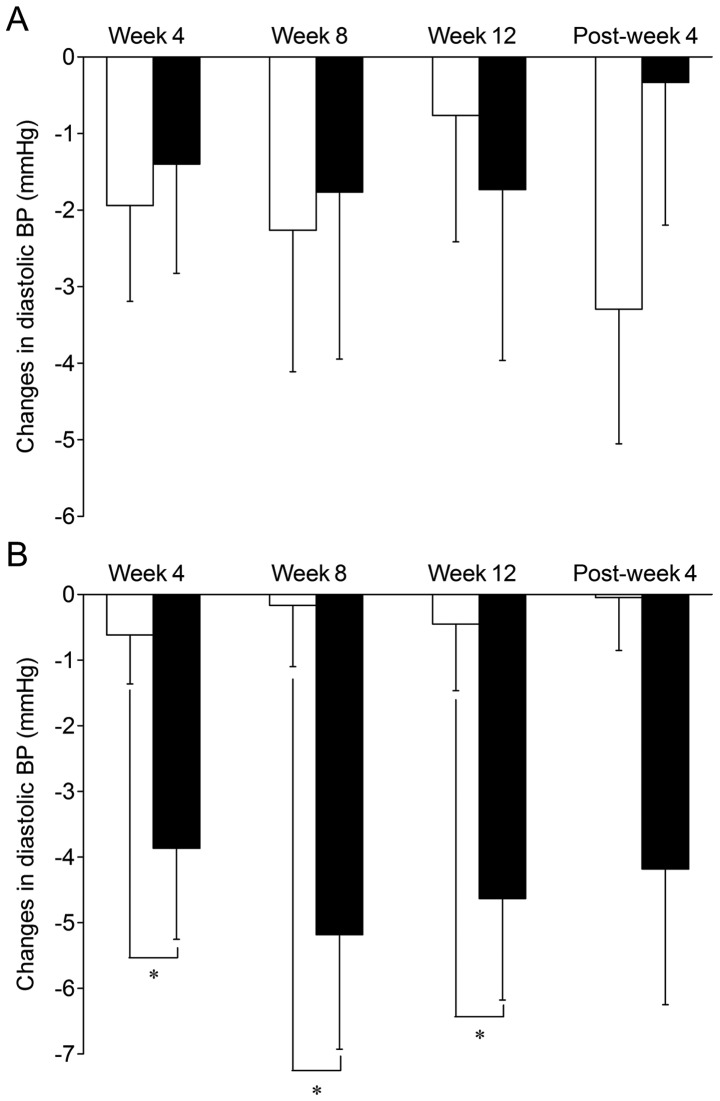
Changes from the baseline diastolic BP following treatment at weeks 4, 8 and 12 and the following treatment at post-week 4 in (A) the placebo group (n=17) and GH group (n=15) of prehypertensive subjects and in (B) the placebo group (n=21) and GH group (n=19) of mildly hypertensive subjects. ^*^P<0.05 against placebo value. White bars represent the placebo groups, and black bars represent GH groups. GH, garlic homogenate-based supplementary diet; BP, blood pressure.

The GH diet treatment also had a hypotensive effect on the diastolic BP in prehypertensive and mildly hypertensive subjects. Table III shows that while the mean diastolic BP for the prehypertensive subjects was not significantly modified by the GH diet treatment throughout the study period, the values for mildly hypertensive subjects were significantly lowered when compared with the baseline at week 4 (4.3%; P<0.05), week 8 (5.8%; P<0.01) and week 12 (5.1%; P<0.01). Fig. 2 shows that between-group differences in the diastolic BP for mildly hypertensive subjects reached significant levels at week 4 (mean change from baseline: GH, −3.9±1.4 mmHg; placebo, −0.6±0.7 mmHg; P<0.05), week 8 (mean change from baseline: GH, −5.2±1.7 mmHg; placebo, −0.2±0.9 mmHg; P<0.05) and week 12 (mean change from baseline: GH, −4.6±1.5 mmHg; placebo, −0.5±1.0 mmHg; P<0.05), while no such significant between-group differences were observed for prehypertensive subjects at any time.

Numerous clinical studies performed in the past 30 years to examine the hypotensive effect of garlic preparations have shown controversial results, as have been summarized in 5 systematic reviews and/or meta-analyses (12–15,17). Whereas several trials have suggested that garlic has possible BP-lowering effects, a larger number of published studies have reported virtually no effect. However, it is notable that all positive studies were performed using hypertensive subjects (baseline systolic BP, ≥140 mmHg) (18–21) and that, by contrast, almost all negative studies used normotensive subjects (systolic BP, <140 mmHg) as the study population (22–26).

Consistent with these existing positive reports, the present study, in which a new garlic-based supplementary diet, the GH diet was used, and showed that it significantly reduced systolic and diastolic BPs in mildly hypertensive subjects following 8- and 12-week treatments, and that, by contrast, the treatment does not impact BP in prehypertensive subjects. This latter finding may reflect a pharmacological phenomenon that has been reported with certain antihypertensive medications; as the BP approaches normal values, antihypertensives have less effect. The type of study population (whether the subjects are normotensive or hypertensive) is crucial in determing the BP-lowering effects of garlic and its preparations, including the GH diet, as suggested in the two meta-analyses (13,14). Although short-term (12-week) treatment with the GH diet had no significant BP-reducing effects in prehypertensive subjects, long-term trials are warranted to examine whether this garlic preparation may be effective in forestalling progression into a hypertensive state, as observed in treatment with certain other antihypertensives (2,27).

The results obtained from the mildly hypertensive subject groups in the present study appear to be in accordance with those from earlier positive placebo-controlled studies demonstrating the significant hypotensive effects of conventional garlic preparations, mainly dried garlic powder products, in individuals who had mild or moderate hypertension (18–21,28). Several meta-analyses of placebo-controlled trials, evaluating the efficacy of commercial, dried garlic powder products in daily doses of 600–900 mg in the treatment of high BP, revealed reductions in systolic BP and diastolic BP of 7–16 and 3–9 mmHg, respectively (12–14). The results of the present study from an 8- or 12-week intake of GH diet, in a daily dose of 300 mg as dehydrated garlic homogenate, showed decreases of 6.6–7.5 mmHg and 4.6–5.2 mmHg from the baseline or placebo in systolic and diastolic BPs, respectively, in subjects with mild hypertension. Therefore, the hypotensive effects of the GH diet do not appear to be dissimilar to those reported for dried garlic powder products. The findings of the present study on the hypotensive effects of the GH diet would have beneficial implications on health at the population level, where a reduction of 4–5 mmHg in systolic BP and 2–3 mmHg in diastolic BP has been estimated to reduce the risk of cardiovascular morbidity and mortality by 8–20% (29).

The mechanisms behind the hypotensive action of the GH diet and the active component(s) involved remain in need of clarification. Our previous animal and *in vitro* studies demonstrated that the GH diet and its major sulfur-containing constituent, γ-glutamyl-S-allyl-cysteine (GSAC), are able to lower BP in hypertensive rats (30). They have the activities required to inhibit angiotensin I-converting enzyme (ACE) and to induce endothelium-dependent and -independent relaxation of the isolated rat aorta (16,31). This leads us to consider the possibility that GSAC with its ACE-inhibitory and vasodilating activities may play a major role in the hypotensive effect of the GH diet in subjects with high BP.

### Effect on hematology and blood chemistry

There were no hematological or blood chemistry parameters that demonstrated a significant change from the baseline in subjects consuming the GH diet over the placebo throughout the study period when analyzed with prehypertensive subjects and mildly hypertensive subjects separately (data not shown). Of all the laboratory parameters, the most prominent change over time was observed for the LDL-cholesterol level in the GH group. When compared with the baseline figures, mean values were decreased by 5.3 mg/dl (4.0%; P>0.05) at week 4, by 7.7 mg/dl (5.7%; P>0.05) at week 8, by 13.1 mg/dl (9.8%; P<0.05) at week 12 and by 6.0 mg/dl (4.5%; P>0.05) at post-week 4.

We observed no significant effects from the 12-week intake of the GH diet versus the placebo on any laboratory parameters, including those correlated with metabolic diseases. However, it remains likely that GH may have a potential for reducing LDL-cholesterol, as the mean values for the GH group were reduced significantly at all times during the 12-week treatment period compared with the baseline figures. With regard to the potential of garlic preparations, particularly the dried garlic powder, for lowering blood levels of total cholesterol and/or LDL-cholesterol, mixed results have been obtained from a large number of prior randomized, double-blind placebo-controlled trials, as observed in a systemic review by Turner *et al*(32) and a meta-analysis by Khoo and Aziz (33). Larger scale, long-term trials are likely to be required to determine whether there is a possible beneficial effect of GH diet on blood cholesterol.

### Safety and tolerability

The GH diet and the placebo were well tolerated. When the whole study population was analyzed, the incidence and pattern of adverse events that occurred throughout the 12-week treatment period and the 4-week follow-up period in the GH group were almost equivalent to those in the placebo group. Only 6 of the 34 subjects (18%) in the GH group and 7 of the 38 subjects (18%) in the placebo group reported minor adverse events, the most common being gastric distress (5 in the GH group and 4 in the placebo group). Less frequent adverse events included headaches (3 each in the GH and placebo groups) and abdominal pain with diarrhea (3 events in the GH group and 1 in the placebo group). All these self-recorded adverse events were extremely mild in intensity, occurred only temporarily and were judged by the investigator as unrelated to the study treatment. It is noteworthy that none of the subjects on the GH diet reported a garlic taste, garlic breath or an unpleasant body odor.

A 12-week treatment with the GH diet had no clinically significant untoward side-effects. It is generally accepted that garlic is safe in a wide range of doses. Only a few studies among a large number of clinical trials have reported the occurrence of adverse events as a result of treatment with garlic preparations. The most frequent event or complaint associated with garlic therapy is a garlic odor on the breath and body, with the next complaints being mild gastrointestinal adverse events, including nausea, bloating and flatulence (34). The results of the present study showed that no subjects treated with the GH diet reported intolerable garlic odor or any other side-effect. Coating the garlic homogenate core with a capsule and/or combining it with egg yolk may contribute to the safety and tolerability of the GH diet.

In conclusion, the results of the present study have demonstrated that a daily 300-mg as dehydrated garlic homogenate dose of the GH diet lowered systolic BP by 6.6–7.5 mmHg and diastolic BP by 4.6–5.2 mmHg in subjects with mild hypertension, but not in those with prehypertension, following an 8- or 12-week treatment. The substantial hypotensive effects of the GH diet appeared to continue for at least 4 weeks following the termination of the 12-week treatment. The GH diet was well tolerated without any clinically significant untoward effects. These results lead us to the conclusion that GH diet may have certain benefits as a complementary therapy for mildly hypertensive subjects.

## Figures and Tables

**Table I.** Baseline characteristics of the randomized, grouped subjects.

	Prehypertensive		Mildly hypertensive	
Parameter	Placebo group (n=18)	GH group (n=16)	Placebo group (n=24)	GH group (n=23)
Age (years)	47±16	53±12	53±9	54±8
Gender (male/female)	11/7	10/6	13/11	13/11
Weight (kg)	64±13	68±13	64±10	62±9
Body mass index (kg/m^2^)	24±4	25±3	25±3	23±2
Systolic/diastolic BP (mmHg)				
Measured at clinic visit	134±3/82±5	134±4/83±6	142±6/92±6	142±6/91±6
Measured at home	138±11/84±6	143±15/86±8	150±12/92±8	149±11/94±9
Blood chemistry				
LDL-cholesterol (mg/dl)	124±34	142±37	128±39	132±26
HDL-cholesterol (mg/dl)	62±15	55±18	61±17	71±19
Triglycerides (mg/dl)	100±54	131±63	124±61	117±48
Fasting glucose (mg/dl)	93±9	92±14	94±9	94±10
HbA1c (%)	5.1±0.3	5.1±0.3	5.1±0.3	5.1±0.3
Dietary composition				
Total energy (kcal/day)	1838±367	1753±239	1911±578	1845±506
Total protein (g/day)	66±19	68±13	71±25	73±18
Total fat (g/day)	59±18	55±17	67±27	63±29
Total carbohydrate (g/day)	249±52	236±40	248±78	237±55
Total mineral (g/day)	9.3±1.2	10.1±2.2	10.1±3.6	10.2±3.3
Exercise-associated energy consumption (kcal/day)^a^	313±160	245±139	240±123	217±102

All values are expressed as the mean ± standard deviation (SD), with the exception of gender. In the prehypertensive and mildly hypertensive populations, there were no statistically significant between-group differences (P<0.05).

a Calculated on the basis of measurements by a passometer. GH, garlic homogenate-based supplementary diet; BP, blood pressure.

**Table II.** Changes in systolic BP measured over the 12-week treatment and 4-week post-treatment follow-up periods in prehypertensive and mildly hypertensive subjects.

	Prehypertensive		Mildly hypertensive	
Time	Placebo group (n=17)	GH group (n=15)	Placebo group (n=21)	GH group (n=19)
Baseline	133.6±0.8	133.5±1.2	142.3±1.2	142.7±1.3
Week 4	129.4±1.6^a^	127.9±1.7^a^	141.2±1.9	137.7±2.2^a^
Week 8	129.7±2.0	131.0±1.7	140.9±1.9	135.2±1.5^b^
Week 12	131.7±1.4	129.9±2.1	141.6±1.6	136.1±1.8^b^
Post-week 4	128.9±1.6^a^	131.9±1.5	141.6±2.0	137.2±1.5^b^

Values are the mean ± standard error of the mean (SEM). Baseline values were determined immediately prior to the start of treatment.

a P<0.05,

b P<0.01 against baseline (within-group differences in measurement). GH, garlic homogenate-based supplementary diet; BP, blood pressure.

**Table III.** Changes in diastolic BP measured over the 12-week treatment and 4-week post-treatment follow-up periods in prehypertensive and mildly hypertensive subjects.

	Prehypertensive		Mildly hypertensive	
Time	Placebo group (n=17)	GH group (n=15)	Placebo group (n=21)	GH group (n=19)
Baseline	82.0±1.1	82.6±1.7	91.2±1.3	90.2±1.6
Week 4	80.1±1.3	81.2±1.3	90.6±1.3	86.3±1.5^a^
Week 8	79.7±2.0	80.9±1.4	91.0±1.6	85.0±1.0^b^
Week 12	81.2±1.6	80.9±1.6	90.8±1.6	85.6±1.5^b^
Post-week 4	78.7±1.8	82.3±1.7	91.2±1.6	86.0±1.6

Values are the mean ± standard error of the mean (SEM). Baseline values were determined immediately prior to the start of treatment.

a P<0.05,

b P<0.01 against baseline (within-group differences in measurement). GH, garlic homogenate-based supplementary diet; BP, blood pressure.
